# Design and Optimization of Chitosan/Montmorillonite Nanoparticle for Sustained Vancomycin Delivery in Peri-implantitis

**DOI:** 10.5812/ijpr-161934

**Published:** 2025-12-07

**Authors:** Shima Esmailzadeh, Taherehsadat Jafarzadeh, Mohammad Moazen

**Affiliations:** 1 Department of Dental Biomaterials, Faculty of Dentistry, Tehran University of Medical Sciences, Tehran, Iran; 2 Department of Pharmacoeconomics and Pharmaceutical Administration, Faculty of Pharmacy, Tehran University of Medical Sciences, Tehran, Iran

**Keywords:** Chitosan, Montmorillonite, Vancomycin, Nanoparticles, Peri-implantitis, Sustained Release, Drug Delivery Systems, Antimicrobial Activity, Cytotoxicity

## Abstract

**Background:**

Dental implants are increasingly utilized to replace lost teeth; however, peri-implantitis — a condition primarily caused by bacterial plaque — poses substantial challenges to maintaining implant success and the health of surrounding tissues. Effective management strategies, including localized antibiotic delivery, are essential.

**Objectives:**

This study aims to investigate an innovative treatment for peri-implantitis utilizing a chitosan-based nanocarrier formulated with montmorillonite and vancomycin (CS/MMT/VAN). The focus is on optimizing the formulation to enhance antibiotic therapy.

**Methods:**

Optimization of various nanoparticle concentrations and ratios was performed using design of experiment (DoE) software to ensure efficient drug delivery. Characterization of the nanoparticles was performed through scanning electron microscopy (SEM), Polydispersity Index (PDI), X-ray diffraction (XRD), and Fourier-transform infrared spectroscopy (FTIR). The nanoparticles were synthesized via electrospray and incorporated into a sol-gel carrier system. Additionally, a thermo-responsive gel was developed to evaluate its gelling properties and potential as a delivery medium. In vitro antimicrobial and cytotoxicity assays were performed.

**Results:**

The study demonstrated nanoparticle sizes ranging from 117 to 389 nm with encapsulation efficiency (EE) between 52% and 88%. The optimized CS/MMT/VAN formulation contained 2.45% CS, a polymer-to-drug (P/D) ratio of 2.21%, and a CS-to-clay ratio of 2.43%. This formulation exhibited a sustained vancomycin (VAN) release profile, characterized by an initial burst followed by prolonged release over 21 days. Characterization confirmed an average particle size of approximately 300 nm and EE close to 85%. In vitro antimicrobial and cytotoxicity assays further validated the efficacy and safety of the formulation.

**Conclusions:**

The developed CS-based nanocarrier demonstrates significant potential in the management of peri-implantitis through its effective drug delivery mechanism. Further clinical evaluation is warranted to ascertain its efficacy in in vivo applications.

## 1. Background

A dental implant is one of the superior treatments to replace missing teeth if mechanical and biological factors are considered (1, 2). The prevalence of dental implants has demonstrated a notable upward trend, with projections suggesting a potential increase to 23% in the future (3). The success of dental implant treatment depends on healthy gingival tissue, the absence of marginal bone loss, and effective osseointegration (4). Peri-implantitis, characterized by plaque accumulation around implants leading to inflammation and bone loss, poses a significant challenge to implant longevity (5). The reported prevalence of peri-implant mucositis and peri-implantitis is approximately 43% and 22%, respectively (6). Therefore, early treatment or prevention of peri-implantitis is of great importance due to the costs involved (5).

*Staphylococcus aureus* is a major contributor to implant treatment failure due to its strong adherence to titanium implant surfaces (5, 7, 8). Vancomycin (VAN) is considered the most effective antibiotic against this pathogen (9). While antibiotics such as cefazolin, nafcillin, oxacillin, daptomycin, and linezolid are commonly employed against staphylococcal infections, VAN remains essential for treating severe cases, especially given the increasing resistance of staphylococcal strains to traditional antibiotics (10). Notably, VAN maintains bactericidal activity by exceeding the minimum inhibitory concentration (MIC) against *S. aureus* even after prolonged treatment periods of up to 16 days (11). Additionally, localized antibiotics such as VAN effectively eradicate peri-implant bacteria and promote osseointegration (12, 13). Therefore, reducing biofilm formation and *S. aureus* presence is critical to improving implant treatment success and osseointegration (14).

Chitosan (CS)-based nanocarriers are widely valued in drug delivery for their biocompatibility, biodegradability, and multifunctional bioactivities, including antibacterial and anti-inflammatory effects (15-17). The CS alone suffers from limitations including pH sensitivity, solubility issues, and variable drug release control (18, 19). Incorporating montmorillonite (MMT) clay into CS matrices enhances mechanical strength, drug encapsulation efficiency (EE), and sustained release compared to conventional carriers. The high surface area and layered structure of MMT create a tortuous path for drug diffusion, enabling controlled and prolonged release, thereby improving delivery performance beyond that of typical CS-based systems (20-25).

Surface response methodology (SRM) is a statistical and mathematical optimization software that models and evaluates the effects of multiple independent variables on desired responses, such as drug release and EE. By systematically analyzing these factors, SRM enables identification of optimal formulation conditions that were previously unclear or unexplored. This approach effectively addresses knowledge gaps by providing a quantitative understanding of the relationships between formulation parameters and performance outcomes. Consequently, SRM reduces dependency on trial-and-error, enhances formulation predictability, and supports the development of robust drug delivery systems with improved efficiency and controlled release profiles (26).

## 2. Objectives

This study addresses a gap in the current literature by systematically optimizing CS-based nanoparticles combined with MMT clay for sustained VAN delivery targeted specifically at peri-implantitis. While prior research has demonstrated the antimicrobial properties of CS and its applications in oral drug delivery (27, 28), these studies often lack precise control over formulation parameters and do not employ rigorous statistical methodologies such as design of experiment (DoE) for optimization (29). Furthermore, existing work mainly focuses on empirical approaches or broader dental applications rather than addressing the unique challenges of peri-implantitis, such as biofilm resistance by *S. aureus* and the need for prolonged, localized antibiotic release. Incorporating MMT clay enhances nanoparticle stability and drug release control, overcoming CS’s limitations related to pH sensitivity and inconsistent release profiles (28). Through the application of DoE, this study quantitatively optimizes key formulation variables, enabling a reproducible and efficient drug delivery platform that advances beyond traditional trial-and-error methods. This approach offers improved antimicrobial efficacy and supports enhanced osseointegration, thus addressing critical limitations identified in recent studies and contributing to the advancement of peri-implant infection management (27-29).

## 3. Methods

### 3.1. Experimental Design

The effective parameters on size and encapsulation efficacy of CS/clay nanoparticles were optimized using central composite design (CCD) with α = 2 by using DoE experiment software (Design Expert 11.1.1). The CCD was applied because it provides nearly as much information as a multilevel factorial design but requires significantly fewer experimental runs. While Box-Behnken design (BBD) typically involves fewer design points than CCD. The CCD includes axial points that often extend beyond the experimental cube. These additional points help capture a more complete response surface, enabling more accurate optimization of the formulation (30). The independent variables in this study included CS concentration (A), the polymer/drug ratio (B), and the concentration of CS/clay (C). The dependent variables were the size of the nanoparticles (Y_1_) and the EE (Y_2_). The design incorporated two replications of the center points. Based on previous studies and preliminary data, a center point value of 3 (w/v %) was selected for CS concentration, polymer-to-drug (P/D) ratio, and clay content. This value was chosen as it represents a balanced midpoint within the evaluated range, facilitating effective optimization and ensuring favorable nanoparticle characteristics such as size, stability, and drug EE (Table 1) (31, 32).

**Table 1.** Factors and Their Levels of Central Composite Design

Parameters	Unit	Notation	Level				
			-α	-1	0	+1	+α
**CS concentration**	%	A	1	2	3	4	5
**Polymer/drug **	Ratio	B	1	2	3	4	5
**CS/clay**	%	C	1	2	3	4	5

Abbreviation: CS, chitosan.

The quadratic model formula for this design is defined as: Y_i_ = b_0_ + b_1_A + b_2_B + b_3_C + b_12_AB + b_13_AC + b_23_BC.

In this context, Y_i_ reflects the measured response, with b_0_ as the intercept and b_1_ to b_23_ as the regression coefficients based on observed experimental values of Y.

### 3.2. Preparation of Chitosan-Clay-Drug Solution

The F1 formulation was prepared by stirring a 3% (w/v) CS solution in 90% (v/v) acetic acid overnight at room temperature. The 90% acetic acid effectively reduces surface tension, increases solution conductivity, and enhances nanoparticle size homogeneity without compromising biocompatibility (31, 33). The CS nanoparticles synthesized in acidic media have been extensively reported as biocompatible and safe, showing low cytotoxicity even without subsequent washing or purification steps. The residual acid content is minimal in the final dried nanoparticles due to rapid solvent evaporation during electrospraying, which greatly reduces acetic acid presence in the particles (34, 35). The MMT solution was prepared by dispersing 1 g clay in 100 µL 90% acetic acid, then 9 mL was added dropwise to the CS solution, followed by VAN at a P/D ratio of 3. The mixture was stirred gently for 24 hours. This procedure was consistently applied to prepare all 16 samples (Table 2). 

**Table 2.** Samples and the Observed Responses of Central Composite Design, Y = Size, and Y_2_ Encapsulation Efficacy ^a, b^

Formulation Codes	Independent Variables			Dependent Variables	
	A ^c^	B ^d^	C ^e^	Y_1_^f^	Y_2_^g^
**F1**	3	3	3	291 ± 17	72 ± 3.7
**F2**	2	2	2	271 ± 19	70 ± 4.8
**F3**	3	5	3	273 ± 20	63 ± 1.6
**F4**	4	2	2	354 ± 9	78 ± 2.7
**F5**	4	4	2	324 ± 11	79 ± 2.1
**F6**	3	3	5	285 ± 21	60 ± 1.5
**F7**	2	4	4	270 ± 24	64 ± 1.4
**F8**	1	3	3	117 ± 17	52 ± 1.1
**F9**	2	2	4	266 ± 23	67 ± 2.3
**F10**	4	4	4	356 ± 8	85 ± 1.4
**F11**	2	4	2	322 ± 14	68 ± 1.8
**F12**	4	2	4	358 ± 18	81 ± 2.8
**F13**	3	3	3	294 ± 11	75 ± 1.1
**F14**	3	1	3	364 ± 29	88 ± 0.9
**F15**	5	3	3	389 ± 19	81 ± 2.6
**F16**	3	3	1	282 ± 15	69 ± 1.6

^a^ The experiments were done in triplicate (n = 16).

^b^ Values are expressed as mean SD.

^c^ Chitosan (CS, %).

^d^ Polymer-to-drug (P/D) ratio.

^e^ C/C ratio.

^f^ Mean ± SD particle size (nm).

^g^ Encapsulation efficacy (%) ± SD.

### 3.3. Viscosity Measurement Method

The viscosity of CS formulations (Table 3) was measured at 25°C using a Ubbelohde capillary viscometer. Samples were filtered through a 0.45 µm membrane before measurement. Flow times were recorded in triplicate to calculate relative, specific, reduced, and intrinsic viscosities, providing insight into rheological properties relevant to nanoparticle formation. In the Supplementary File, detailed descriptions of the viscosity measurement procedures are provided.

**Table 3.** Viscosity of Chitosan-Clay-Drug Solution ^a^

Formulations	CS % (A)	Estimated Viscosity (mPa × s) Range
**F8**	1	~ 200 - 400
**F2, F7, F9, F11**	2	~ 400 - 800
**F1, F3, F6, F13, F14, F16**	3	~ 500 - 1500
**F4, F5, F10, F12**	4	~ 1000 - 2000
**F15**	5	~ 1500 - 2500

Abbreviation: CS, chitosan.

^a^ The experiments were done in triplicate.

### 3.4. Conductivity Measurement Method

Conductivity measurements were performed at 25°C using a calibrated digital conductivity meter (Table 4). Samples were equilibrated to room temperature and homogenized prior to measurement. Each measurement was conducted in triplicate with standard electrode cleaning between samples. In the Supplementary File, detailed descriptions of the conductivity measurement procedures are provided.

**Table 4.** Conductivity of Chitosan-Clay-Drug Solution ^a, b^

Formulation Codes	CS (%)	P/D Ratio	Clay/CS Ratio	Conductivity (µS/cm)
**F1**	3	3	3	1200 ± 15
**F2**	2	2	2	1050 ± 12
**F3**	3	5	3	1300 ± 18
**F4**	4	2	2	1400 ± 14
**F5**	4	4	2	1500 ± 16
**F6**	3	3	5	1250 ± 15
**F7**	2	4	4	1100 ± 10
**F8**	1	3	3	1000 ± 11
**F9**	2	2	4	1080 ± 12
**F10**	4	4	4	1550 ± 17
**F11**	2	4	2	1120 ± 14
**F12**	4	2	4	1450 ± 15
**F13**	3	3	3	1220 ± 15
**F14**	3	1	3	1180 ± 10
**F15**	5	3	3	1600 ± 20
**F16**	3	3	1	1190 ± 11

Abbreviations: CS, chitosan; P/D, polymer-to-drug.

^a^ The experiments were done in triplicate.

^b^ The values are expressed as mean ± SD.

### 3.5. Preparation of Nanoparticles

The prepared solutions listed in Table 2 were subjected to electrospray using a Fanavaran Nano-Meghyas (Tehran, Iran) system under the following conditions: A flow rate of 0.2 mL/h, an applied voltage of 20 kV, a needle diameter of 1.27 mm, and a tip-to-collector distance of 80 mm. The aerosols generated were collected on aluminum foil substrates measuring 3 × 3 cm^2^.

### 3.6. Scanning Electron Microscopy

The surface morphology of 16 samples was studied with a Hitachi IB-2 coater, followed by palladium coating evaluation using a JEOL JXA-840A scanning electron microscope. The formulations F1 (Figure 3 in the Supplementary File) and F8 (Figure 4 in the Supplementary File), which were studied using scanning electron microscopy (SEM), are detailed in the Supplementary File as non-optimized formulations. The SEM of the optimized formulation is shown.

### 3.7. Dynamic Light Scattering and Polydispersity Index

Dynamic light scattering (DLS) and Polydispersity Index (PDI) measurements were performed using a Horiba SZ-100 instrument. Sixteen samples were dispersed in 5 mL of phosphate-buffered saline (PBS, pH 7.4), sonicated for 5 minutes, and loaded into the instrument cuvette. Measurements were conducted in triplicate at 25°C, and average particle sizes and PDI values were reported. The formulations F1 (Figure 1 in the Supplementary File) and F8 (Figure 2 in the Supplementary File), which were studied using DLS, are detailed in the Supplementary File as non-optimized formulations.

### 3.8. Encapsulation Efficiency of Nanoparticles

**Figure 1.**
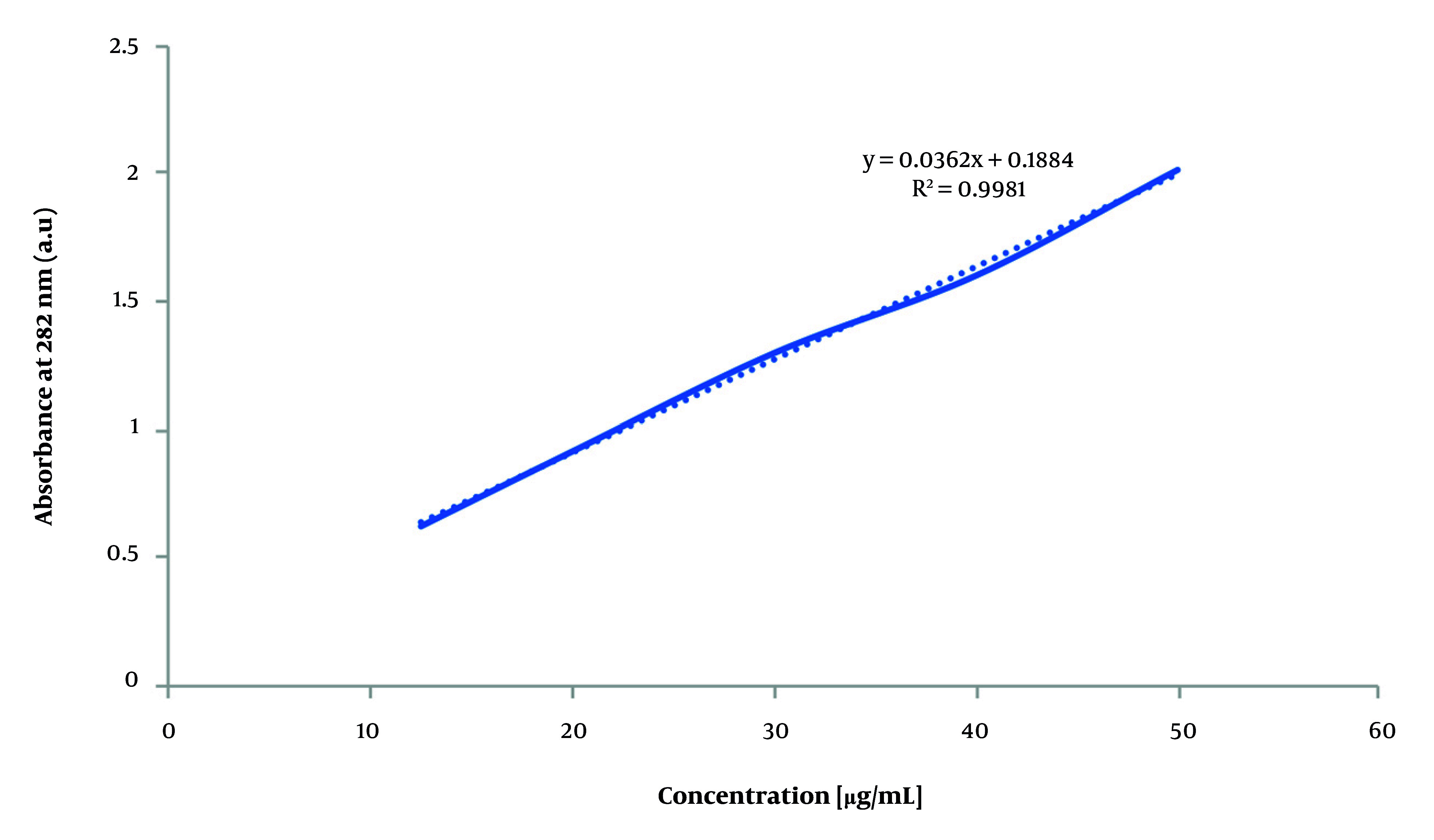
Calibration curve of vancomycin (VAN)

To evaluate VAN EE, 0.2 mL of solution was electrosprayed onto 3 × 3 cm^2^ aluminum foil and dissolved in 5 mL distilled water using a 30-minute ultrasonic bath. The solution was filtered through a 0.22 µm syringe filter, and unencapsulated drug concentration was determined by UV-Vis spectroscopy at 282 nm (Shimadzu UV-1800, Japan) (36). Concentrations were calculated using the calibration curve Y = 0.0362X + 0.1884 (Figure 1), and EE was subsequently calculated using the formula: Encapsulation efficiency (%) = [(Total drug - Free drug) / Total drug] × 100 (37).

### 3.9. Zeta Potential

Zeta potential of the optimized formulation was measured at 25°C using the Horiba SZ-100 instrument with samples prepared in PBS (pH = 7.4).

### 3.10. Preparation of Thermo-Responsive In-Situ Gel with Nanoparticles

To prepare the thermo-responsive gel, stir 18% w/v poloxamer 407 in distilled water overnight at 5°C, then add the optimized formulation and stir for 4 hours at the same temperature to ensure proper gel preparation and nanoparticle distribution.

### 3.11. Determination of Gelling Temperature, Viscosity, and pH of the In-Situ Gels

The gelling temperature of the solution was measured using the vial tilting method. A 1 mL sample was heated from 20°C to 40°C at a rate of 1°C/min. At each temperature increment, the vial was tilted 90° and observed for one minute; the gelling temperature was recorded when no flow was observed upon tilting (38). Viscosity measurements were conducted at 5°C and 37°C using a Brookfield DVTE viscometer (AMETEK, Massachusetts, USA). The pH of the formulation was measured using a calibrated pH meter. All experiments were performed in triplicate to ensure accuracy.

### 3.12. X-ray Diffraction

The samples were characterized by X-ray diffraction (XRD) analysis using an Inel EQ 3000 diffractometer (France) to investigate the intercalation between MMT layers facilitating nanoparticle formation. The XRD is an effective technique for determining crystalline structures, particle size, and intercalation behavior. The diffraction patterns were recorded at 40 kV with Cu-Kα radiation (λ = 1.54 Å) over a 2θ range of 2° to 60°, with a scanning speed of 2°/min. The d-spacing of MMT sheets, affected by CS intercalation, was calculated using Bragg's law, which relates d-spacing to the diffraction angle (θ) and the X-ray wavelength (λ). The formula is as follows: d = λ / [2 sin(θ)]. This analysis allowed for the determination of the spacing of the MMT sheets and the degree of dispersion in the polymer matrix.

### 3.13. Fourier-Transform Infrared Spectroscopy

The ATR-Fourier-transform infrared spectroscopy (FTIR, NICOLET iS10) analyzed functional groups in CS, clay, and CS-clay composites, recording spectra from 4000 to 400 cm^-1^ at a resolution of 4 cm^-1^.

### 3.14. In vitro Drug Releasing of In-Situ Gels

In vitro release of VAN from CS and chitosan/montmorillonite (CS/MMT) nanoparticles was evaluated in PBS (pH = 6.4) incubated at 37°C and 75 rpm using an orbital shaker, reflecting the mean pH of peri-implant crevicular fluid (6.46) (36). Aliquots of the release media were collected at predetermined intervals (2, 4, 8, 24, 48, 72, 168, 336, and 504 hours) and replaced with fresh PBS to maintain a constant volume. The concentration of VAN released was quantified by UV-Vis spectroscopy (39). Release kinetics were analyzed using multiple models to elucidate the drug release mechanism.

### 3.15. Antimicrobial Activity

Gram-positive *S. aureus* (RSKK 1009) was activated from frozen stocks and cultured to 4 × 10^7^ CFU/mL in tryptic soy broth, incubated at 37°C for 24 hours for antimicrobial activity tests. Bacterial cultures were incubated in 6-well plates with serial dilutions of antibiotic solutions for 24 hours on an orbital shaker at 55 rpm and 37°C to assess whether the released antibiotic concentration was sufficient to inhibit bacterial growth. Following this, antibiotic-treated bacterial suspensions were plated on agar and incubated at 37°C for another 24 hours. The MIC was determined by the absence of bacterial colonies on plates.

Antimicrobial activity of the drug-release media from CS/MMT nanospheres was evaluated using the agar diffusion method. The VAN and chlorhexidine discs served as positive controls, while blank discs served as negative controls. Samples (10 μL) were applied on discs at 6 hours, 24 hours, and 21-day intervals (n = 3). Plates were incubated at 37°C overnight, and zones of inhibition were measured after 24 hours.

### 3.16. Evaluating the Cytotoxicity of a Nanoparticle-In-situ Gel

The MTT assay was conducted on isolated gingival fibroblasts in 96-well plates to measure cell viability, with results expressed as a percentage of the control after optical density assessment at 570 nm (15). Human gum fibroblast cells (the Iranian Biological Resource Center in Tehran, Iran), the third to fourth passage, were cultured in controlled conditions and seeded in 96-well plates at a density of 10 × 10^6^ cells per well, and the cells were then incubated for 24 hours. The indirect toxicity of CS/MMT nanoparticle Thermo-gel was assessed by adding cell culture medium at 200 mg/mL (ISO 10993-12), with exposure durations of 24 hours, 5 days, and 21 days. 0.25% (w/v) zinc dibutyldithiocarbamate (ZDBC) were used as positive controls (ISO 10993-12) (40). In this method, the cells were pretreated with eluents at 1:1, 1:4, and 1:16 concentrations. The viability of the cultured cells was analyzed using the MTT assay. After 48 hours of incubation, the supernatant was removed, and 50 µL of MTT solution (5 mg/mL) was added, followed by a further incubation period of 3 - 4 hours at 37°C and 5% CO_2_. The MTT solution was then removed, and 60 µL of DMSO solution was added to the wells. The absorbance was measured at 570 nm using an ELISA reader. The viability of the treated group was reported as a percentage of the control group, which was set at 100%.

## 4. Results and Discussion

### 4.1. Experimental Design

A study formulated 16 samples of CS/clay/VAN nanoparticles using a surface response method, including two center point runs to assess the impacts of three independent variables (A, B, and C) on two response variables (Y_1_ and Y_2_). Each sample was replicated three times, and the nanoparticles' size (Y_1_) and encapsulation efficacy (Y_2_) were evaluated. The average of each run is shown in Table 2. 

#### 4.1.1. Effect of Critical Formulation Factors on the Nanoparticle Size

This study employed regression analysis to model nanoparticle size (Y_1_) based on three critical formulation factors (A, B, and C) from 16 runs generated by SRM for the CS-based nanocarrier formulated with montmorillonite and vancomycin (CS/MMT/VAN). The quadratic formula relating the size of nanoparticles to the three critical factors, expressed in coded form, is presented: Y_1_ = 306.62 + 50.43A - 9.93B + 0.93C - 10.87AB + 11.62AC - 2.37BC.

The formula presented describes a multiple linear regression model predicting the dependent variable Y_1_, using independent variables A, B, and C, along with their interactions. The intercept is set at 306.62, indicating Y_1_'s expected value when all independent variables are zero. The coefficients reveal the influence of the variables on Y_1_, with A showing a significant positive effect (50.43) while B and C exhibit negative impacts. Moreover, the analysis of nanosphere size in distilled water at pH = 7.4 shows a low PDI below 0.25, indicating a uniform size distribution, with an average hydrodynamic size of 306.62 nm. The DLS results suggest that incorporating drugs increases the hydrodynamic size of the nanospheres due to the added volume from drug molecules.

**Figure 2.**
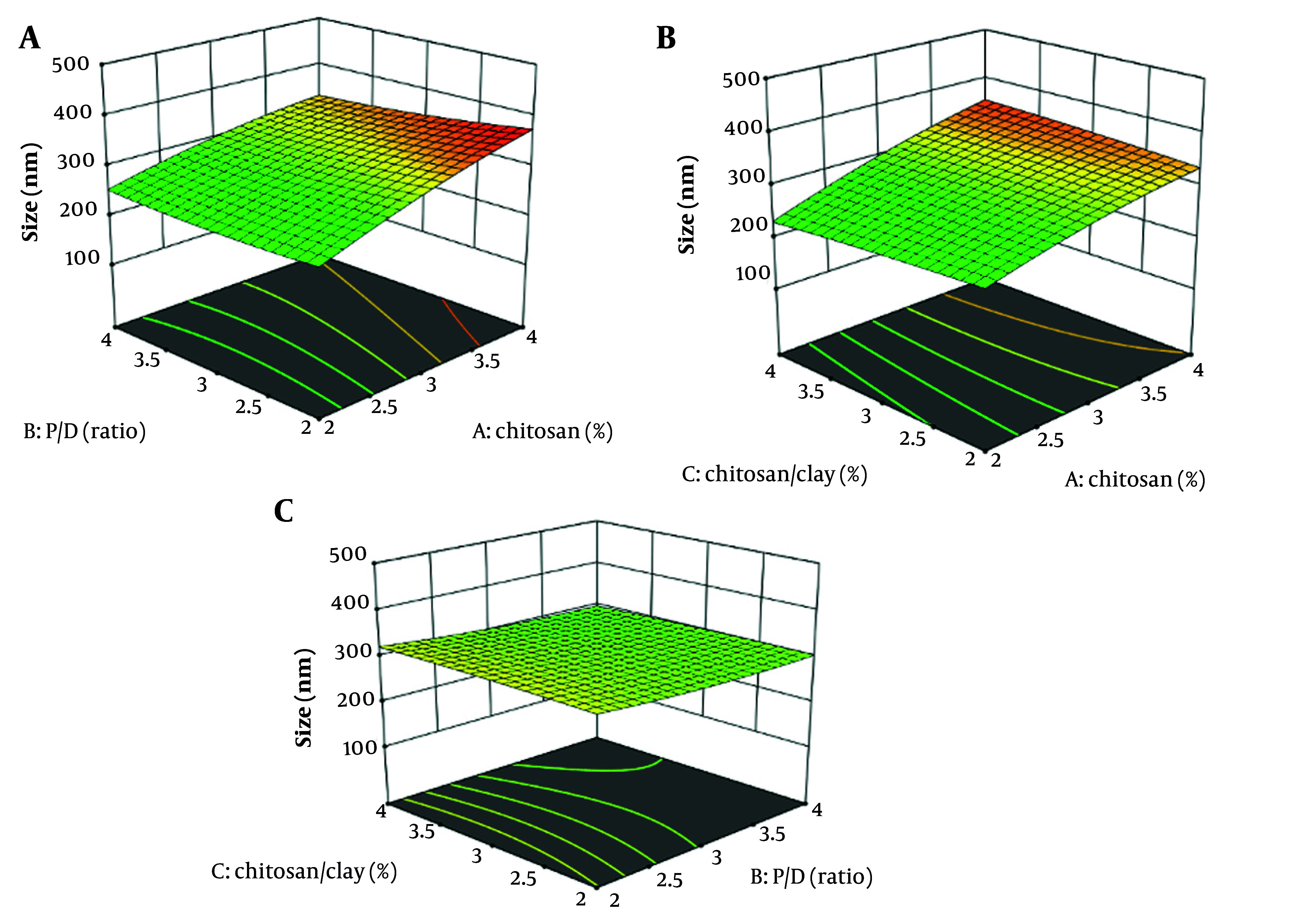
3D response surface plots showing the effect of chitosan (CS, %) (A), and polymer-to-drug (P/D) ratio on size (B), CS (%) and CS/clay (%) on size (C)

**Figure 3.**
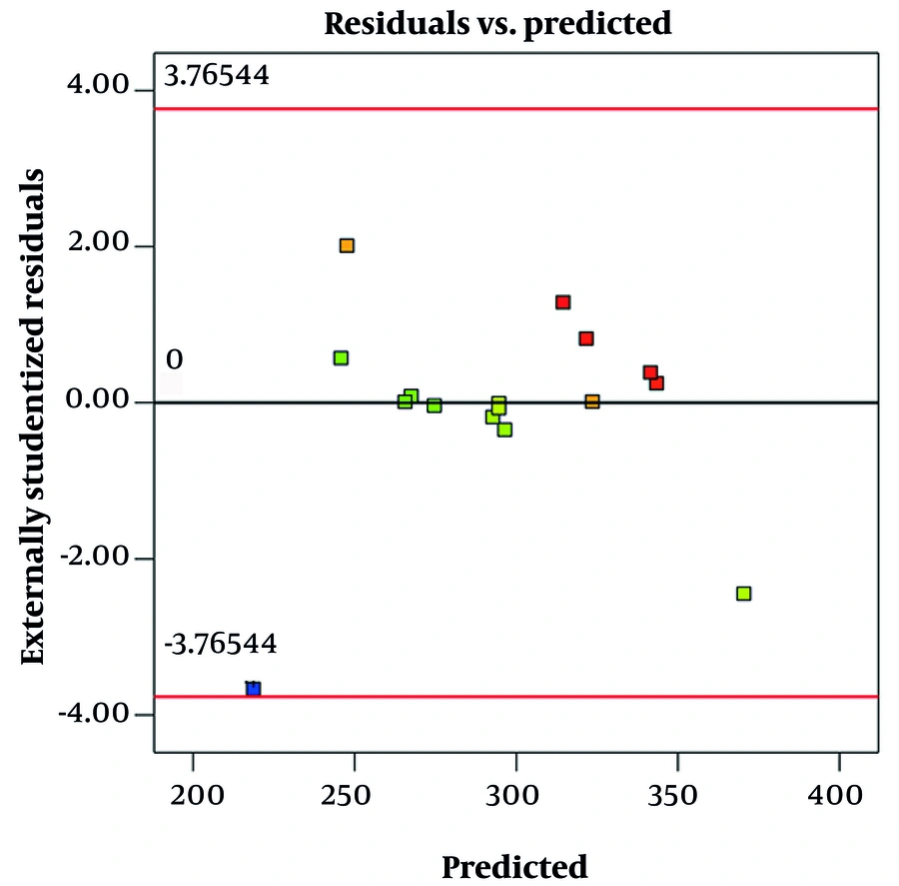
Residual vs. predicted plots for size

The graph in Figure 2A demonstrates that increasing CS concentration results in larger nanoparticles (P < 0.05). However, this larger size can negatively affect encapsulation efficacy, as a decreased surface area limits the number of active ingredients that can be incorporated (Figure 3). Therefore, it is essential to carefully consider the relationship between CS amount, nanoparticle size, and EE (16, 41, 42).

Increased clay concentration in CS solutions does not alter nanoparticle size (P > 0.05) but enhances encapsulation efficacy (43, 44). The clay particles serve as stabilizers, preventing nanoparticle aggregation and improving their surface area, which leads to more effective encapsulation of target molecules (45, 46). Additionally, these clay particles provide a protective barrier, safeguarding nanoparticles from external degradation (47). The MMT enhances EE by intercalating drug molecules into its layered structure, which increases drug loading. It also slows drug diffusion by creating a tortuous path within the polymer matrix, effectively prolonging the release rate (48).

**Figure 4.**
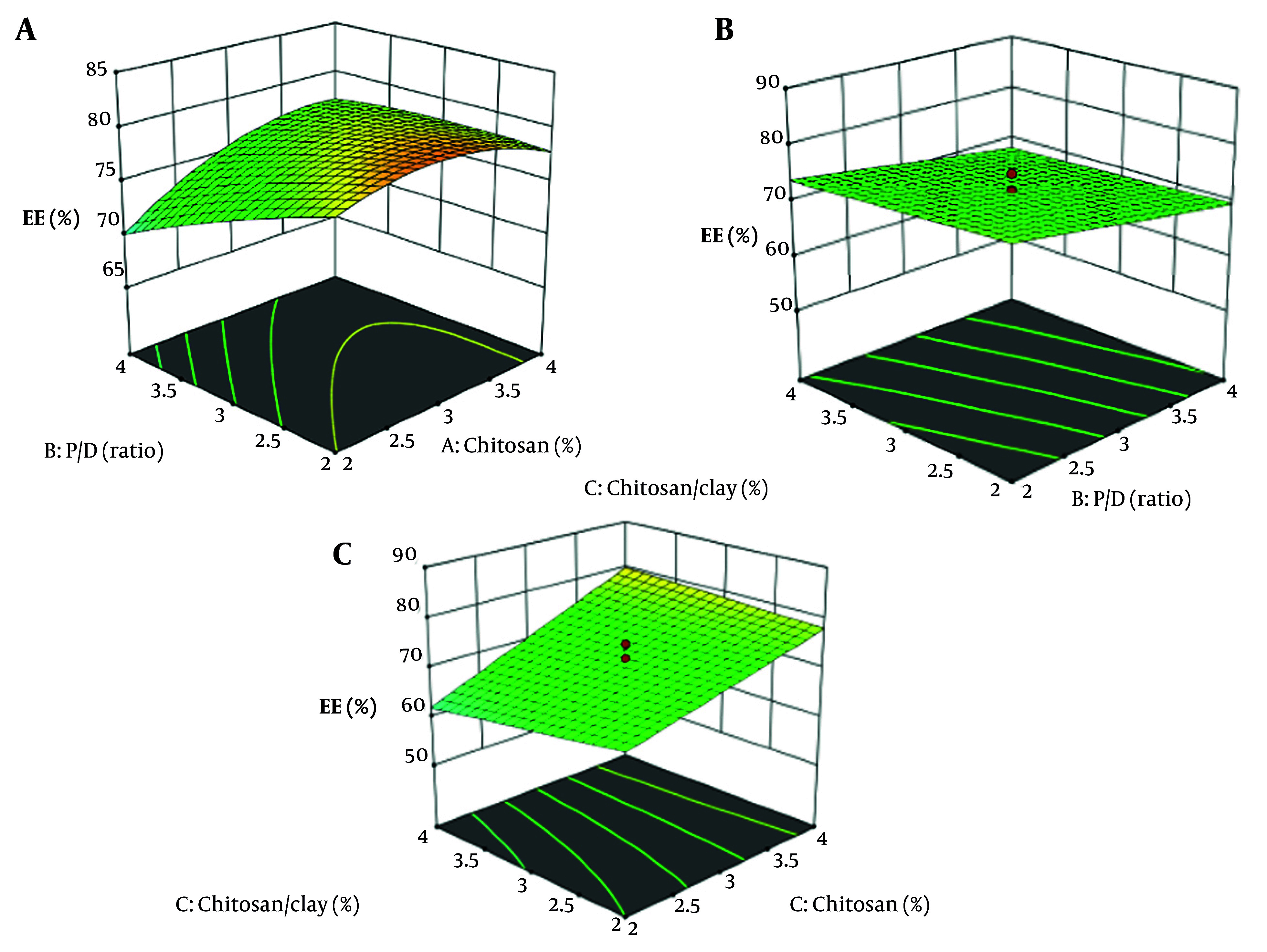
3D response surface plots showing the effect of P/D ratio and Chitosan/Clay (%) on Encapsulation Efficacy (EE) (A), Chitosan (%) and P/D ratio on Encapsulation Efficacy (EE)(B), Chitosan (%) and Chitosan/Clay (%) on Encapsulation Efficacy (EE) (C)

The graphs in Figures 2A and C depict the relationship between the P/D ratio and nanoparticle size, revealing that higher drug loading is associated with increased nanoparticle size (P > 0.05). In contrast, Figure 2B indicates that elevated chitosan concentrations, which also increase nanoparticle size, do not significantly improve drug encapsulation efficiency (Figure 4). The primary determinant of encapsulation efficacy appears to be the composition of the drug carrier matrix, particularly chitosan and clay (16).

In this study, the model explaining the size of CS/clay/VAN nanoparticles showed an R^2^ value of 0.8, indicating that 80% of the variability in particle size was accounted for by the three independent variables (A, B, and C). However, the adjusted R^2^ was lower at 0.52, suggesting that when considering the number of predictors and sample size, the model’s explanatory power is moderate, and some variables may have limited influence. The lack-of-fit test yielded a P-value of 0.11, which is greater than 0.05, indicating no significant lack of fit. This suggests that the model adequately represents the relationship between the independent variables and particle size, and the data fits the model well.

Figure 3 shows a residual vs. predicted plot for size. This graph is used to evaluate the fit and validity of a regression model, specifically how well the model's predicted particle sizes match the observed data. In this plot, the residuals (differences between observed and predicted sizes) are plotted on the y-axis, while the predicted sizes are on the x-axis. Ideally, if the model fits well and assumptions are met, the residuals should be randomly scattered around the horizontal line at zero, with no clear pattern. This randomness indicates constant variance (homoscedasticity) and linearity, suggesting the model is appropriate, which is observed in this graph.

#### 4.1.2. Effect of Critical Formulation Factors on the Encapsulation Efficacy

Encapsulation efficacy plays a crucial role in the formulation of CS/MMT/VAN nanospheres. To explore the relationship between encapsulation efficacy and the three key formulation factors (A, B, and C), a regression analysis was conducted. The quadratic formula derived from 16 experimental runs revealed significant relationships connecting nanoparticle efficacy with the identified factors. This formula can be used to predict the encapsulation efficacy of the nanoparticles based on the three critical factors, in coded form, given: Y_2_ = 72 + 7A - 3.125B - 1C + 1.25AB + 2AC + 0.25BC.

Y2 is a formula that calculates the dependent variable Y2 based on three independent variables — A, B, and C — along with their interactions (AB, AC, BC). The formula includes a constant of 72 and specific coefficients: A has a coefficient of 7, meaning a one-unit increase in A leads to a seven-unit increase in Y_2_, whereas B has a coefficient of -3.125, indicating that a one-unit increase in B results in a three-unit decrease in Y_2_.

Increasing the concentration of CS significantly improves the encapsulation efficacy of the nanoparticles (Figure 4 P < 0.05). This enhancement is attributed to the higher availability of CS chains, which facilitates better drug entrapment within the nanoparticle matrix (16, 49). In contrast, variations in the CS-to-clay ratio and the P/D ratio did not have a statistically significant impact on encapsulation efficacy (P > 0.05) because these factors either do not substantially alter the interaction sites available for drug entrapment or the structural properties of the nanoparticle matrix in a way that would improve drug loading. For example, clay might serve more as a filler or stabilizer rather than directly trapping the drug, so variations in its proportion may have less influence. Additionally, if the P/D ratio is already within an optimal range, further changes might not yield noticeable effects on EE (16).

**Figure 5.**
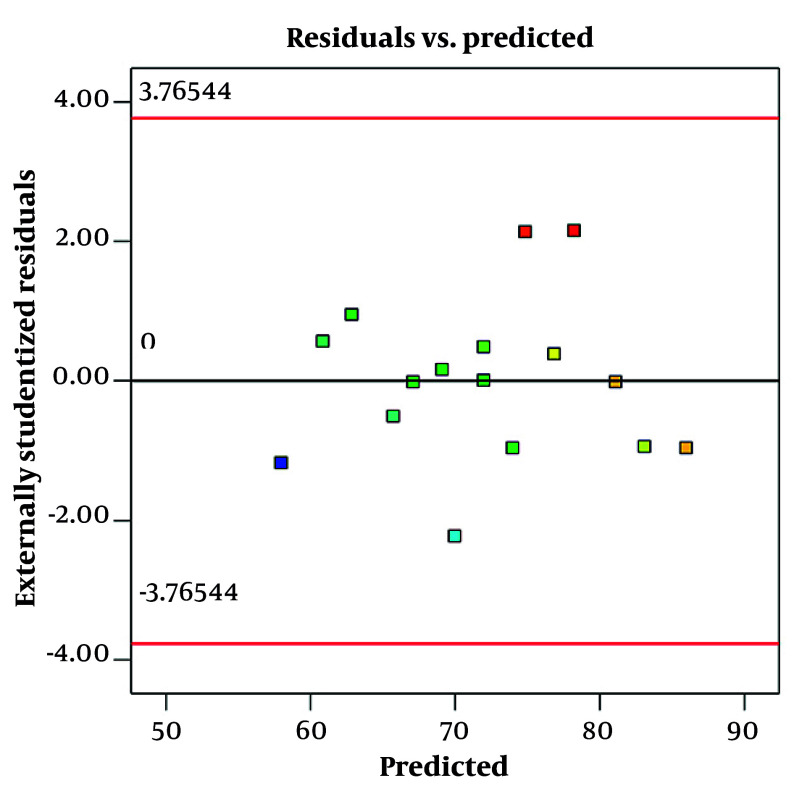
Residual vs. predicted plots for particle size

A residual vs. predicted plot for encapsulation efficacy is a diagnostic tool used to visually assess how well a regression model predicts encapsulation efficacy values (Figure 5). The plot displays residuals — the differences between observed encapsulation efficacy and model-predicted encapsulation efficacy — on the y-axis against the predicted values on the x-axis. This randomness indicates constant variance (homoscedasticity) and linearity, suggesting the model is appropriate, which is observed in this graph.

### 4.2. Viscosity Measurement

Viscosity tends to increase with CS concentration; thus, 1% CS solutions may have lower viscosity near 200 - 400 mPa × s, while 4 - 5% solutions may be in the range of 1000 - 2000 mPa × s or higher, depending on molecular weight and solution conditions. These data are presented in Table 3. 

### 4.3. Conductivity Measurement

The conductivity of CS/clay formulations ranged from approximately 1000 to 1600 µS/cm, increasing with CS and clay content. Higher conductivity improves ionic strength and charge density, enhancing electrospray jet stability and resulting in smaller, more uniform nanoparticles. This aligns with previous findings showing that ionic conductivity is crucial for controlling nanoparticle size and morphology in CS-based systems. The observed conductivity trends correlate with EE, suggesting that optimized ionic environments promote improved drug loading and nanoparticle stability. These data are presented in Table 4. 

### 4.4. Selecting Optimized Formulation

This study utilizes response surface methodology to determine the optimal formulation parameters for producing nanoparticles that achieve high EE while maintaining a small size. The optimal composition was found to be 2.45% CS, a P/D ratio of 2.21, and a CS-to-clay ratio of 2.43.

### 4.5. Zeta Potential and Polydispersity Index

All antibiotic-loaded nanoparticles exhibited a positive zeta potential, beneficial for antibiotic delivery as it prevents aggregation and enhances attachment to negatively charged bacterial cell membranes (50). Positive zeta potential on nanoparticles enhances bacterial adhesion through electrostatic attraction between the positively charged surface and negatively charged bacterial membranes. Studies show that bacterial attachment increases as surface charge shifts from negative to positive, driven mainly by electrostatic forces rather than bacterial viability (51), (52). Additionally, positive zeta potential can disrupt bacterial membranes by neutralizing surface charge and increasing permeability, potentially causing membrane depolarization and cell death. This dual effect supports both strong bacterial binding and antimicrobial activity (50), (53). The 16 nanospheres also demonstrated a low PDI value of less than 0.25, indicating a uniform system with a narrow size distribution (Table 5). 

**Table 5.** Polydispersity Index Value ^a, b^

Formulation Codes	PDI
**F1**	0.23 ± 0.02
**F2**	0.17 ± 0.01
**F3**	0.14 ± 0.01
**F4**	0.15 ± 0.01
**F5**	0.17 ± 0.01
**F6**	0.21 ± 0.02
**F7**	0.18 ± 0.02
**F8**	0.16 ± 0.01
**F9**	0.13 ± 0.01
**F10**	0.25 ± 0.02
**F11**	0.17 ± 0.01
**F12**	0.15 ± 0.02
**F13**	0.14 ± 0.01
**F14**	0.18 ± 0.02
**F15**	0.16 ± 0.01
**F16**	0.20 ± 0.01

Abbreviation: PDI, Polydispersity Index.

^a^ The experiments were done in triplicate (n = 3).

^b^ The values are expressed as mean ± SD.

### 4.6. Determination of Gelling Temperature, Viscosity, and pH of the In-Situ Gels

The gelling temperature of the formulated solution was determined to be 32.5 ± 0.4°C, indicating a sol-to-gel transition near physiological temperature, which is desirable for in-situ gel applications. Viscosity measurements showed values of 45 ± 3 cP at 5°C and 350 ± 15 cP at 37°C, demonstrating a significant increase in viscosity upon heating consistent with gel formation. The pH of the formulation was measured as 6.8 ± 0.1, indicating the formulation is within a suitable range for biological compatibility. All measurements were performed in triplicate to ensure accuracy and reproducibility.

### 4.7. Scanning Electron Microscopy

**Figure 6.**
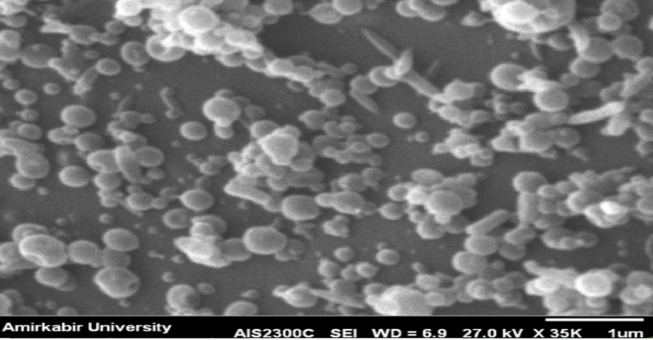
The scanning electron microscopy (SEM) of the optimized formulation of the nanoparticle

The SEM analysis of nanoparticles reveals that the optimized formulation resulted in smooth, spherical nanospheres, enhancing stability and dispersion for drug delivery (Figure 6) (54). Studies demonstrate that nanoparticle size and shape significantly influence cellular uptake and toxicity (55, 56).

### 4.8. X-ray Diffraction of the Optimized Formulation

**Figure 7.**
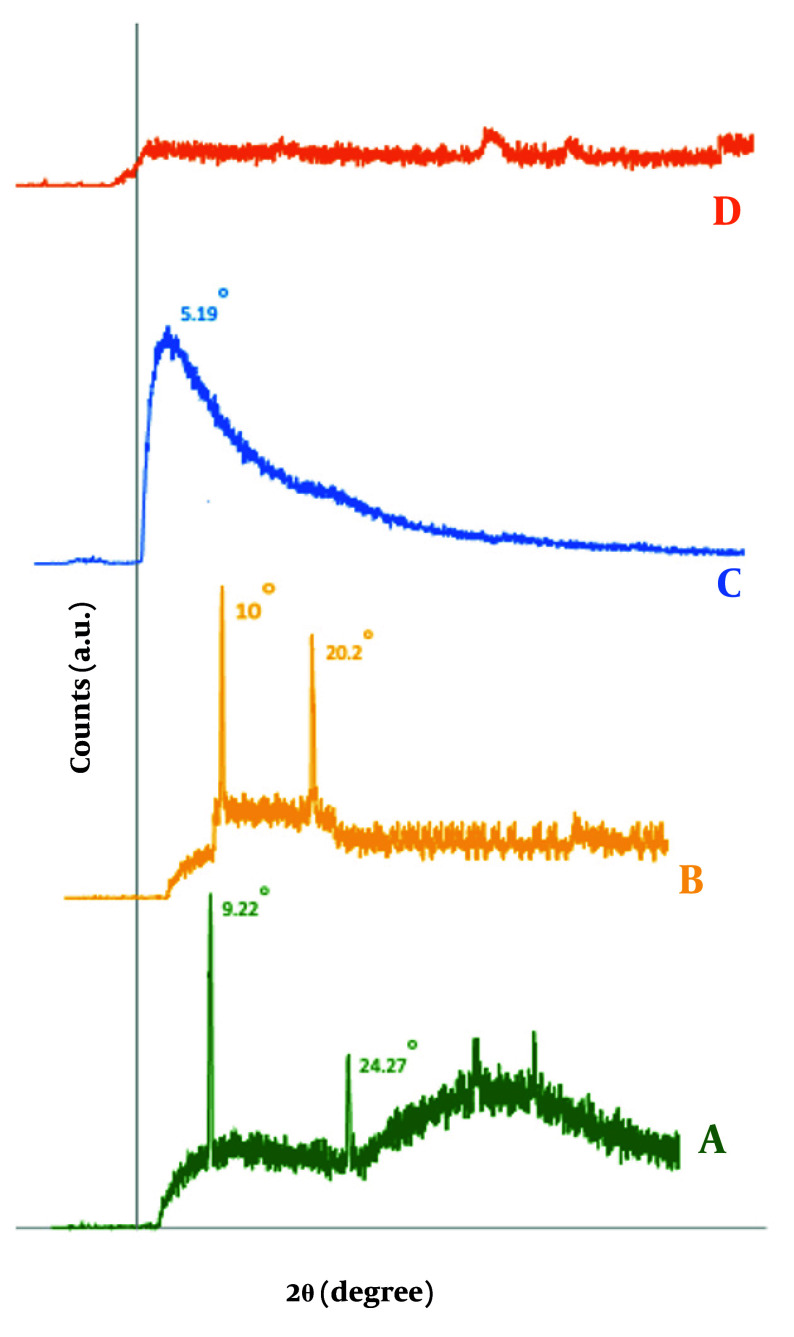
X-ray diffraction (XRD) pattern (A), montmorillonite (MMT) (B), chitosan (CS) (C), freeze-dried CS-MMT (D) CS-MMT nanoparticle

The XRD analysis examined the d-spacing of MMT sheets in CS/MMT nanoparticles. Based on studies, CS exhibited a broad peak at 2θ = 20° and a sharp peak at 2θ = 22° (22), while MMT presented a broad peak at 2θ = 10° and a sharp peak at 2θ = 11° (57). In this study, the MMT diffraction peak was identified at 2θ = 9.25° with a d-spacing of 9.625 Å, which shifted to 2θ = 5.19° and a d-spacing of 17.11 Å for CS-MMT, indicating intercalation due to polymer inclusion (Figure 7). The absence of characteristic peaks in the XRD pattern of CS-MMT nanospheres suggests effective exfoliation of MMT nanoparticles within the CS matrix, although the smaller nanoparticle size may have contributed to the broadening or shifting of diffraction peaks, resulting in undetectability (58).

### 4.9. Fourier-Transform Infrared Spectroscopy of the Optimized Nanoparticle

**Figure 8.**
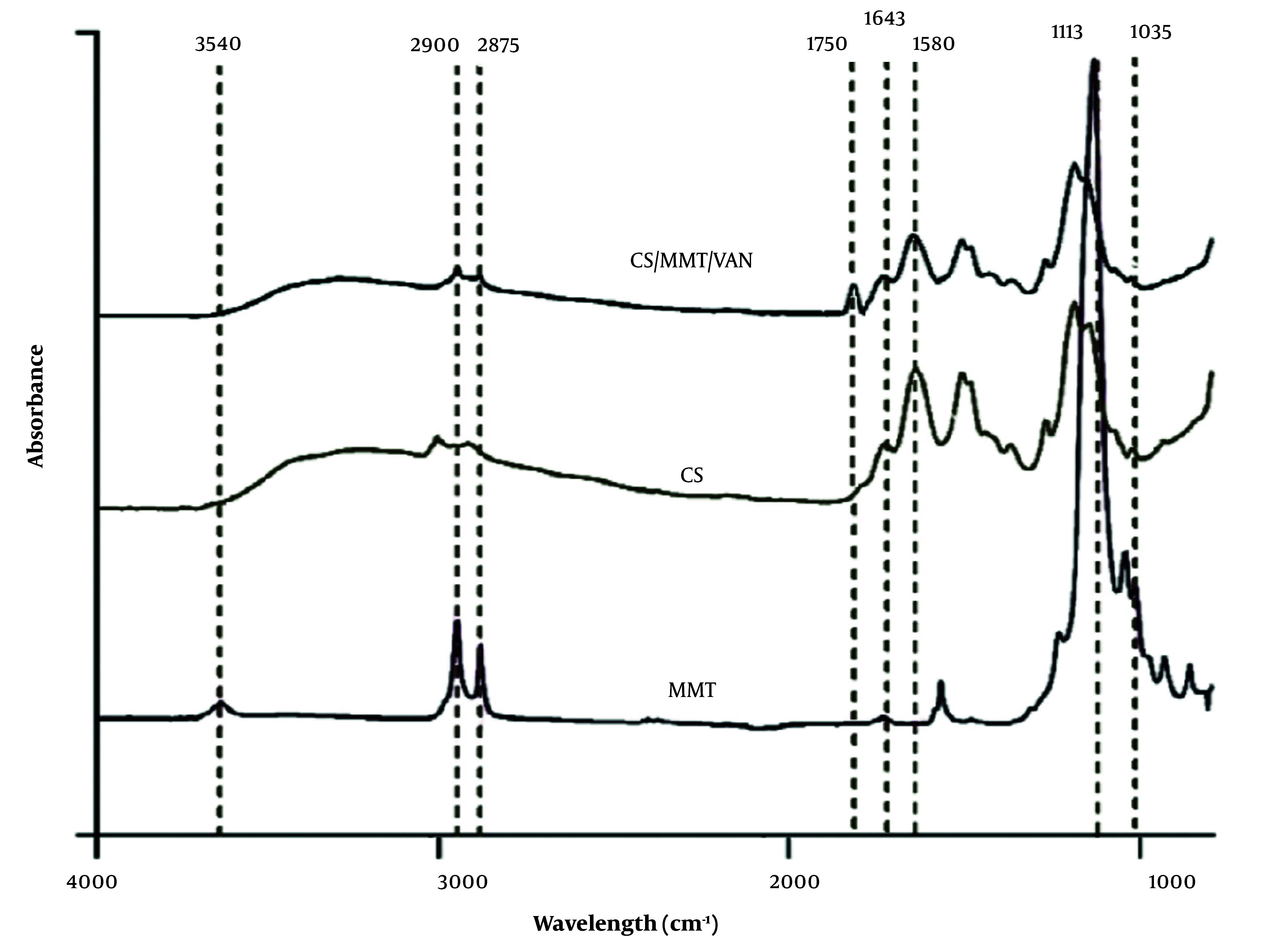
Fourier-transform infrared spectroscopy (FTIR) analysis of montmorillonite (MMT), chitosan (CS), and CS-based nanocarrier formulated with montmorillonite and vancomycin (CS/MMT/VAN)

The FTIR analysis provides significant insights into the interactions within CS, MMT, and VAN composites. The CS spectrum displays characteristic peaks indicating functional groups such as N–H and hydroxyl at 3450 cm^-1^, C–H stretches at 2900 and 2875 cm^-1^, and distinct amide vibrations at 1643, 1580, and 1320 cm^-1^ (59). For MMT (Figure 8), key bands are observed for O–H stretching (3440 - 3620 cm^-1^) and Si–O stretching (1113 - 1035 cm^-1^) (60), while shifts in the amine peaks of the CS-MMT (Figure 8) nanoparticles suggest interactions between CS and MMT (25). The VAN spectrum shows a strong C=O band at 1650 cm^-1^ along with other characteristic signals (61). In the CS/MMT/VAN nanospheres, the FTIR spectrum indicates successful intercalation of VAN, evidenced by shifts in amide and hydroxyl bands along with a new peak at 1750 cm^-1^, reflecting the interactions between the drug and the nanoparticles (39).

### 4.10. In vitro Drug Release of in-Situ Gels

**Figure 9.**
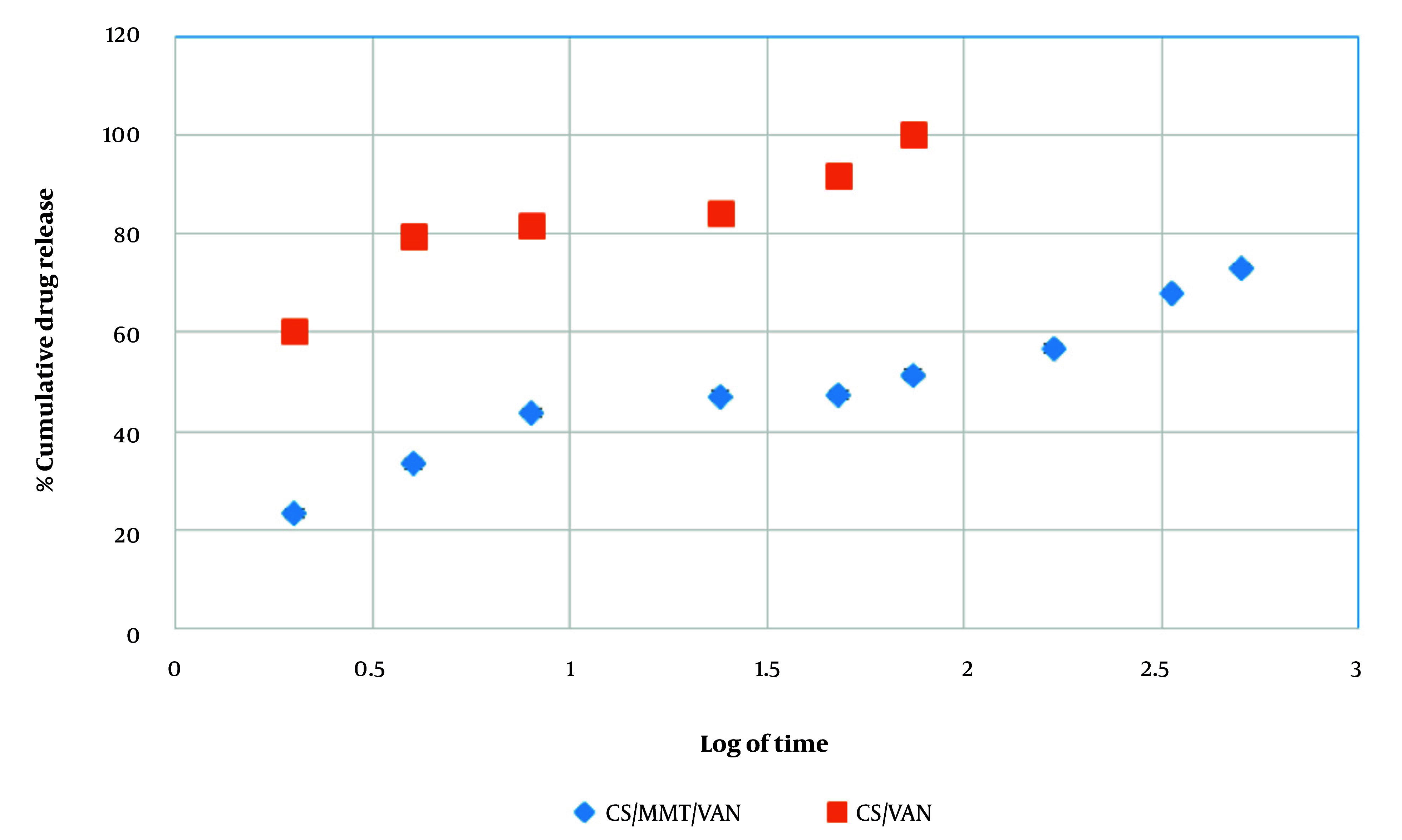
Cumulative drug release of the chitosan/vancomycin (CS/VAN) and chitosan (CS)-based nanocarrier formulated with montmorillonite and vancomycin (CS/MMT/VAN) nanoparticle versus immersion time

This study evaluated the effect of MMT incorporation on the drug release profile of CS nanoparticles. The control group — pure CS nanoparticles — exhibited a limited release, with VAN release confined to the initial 4 hours and characterized by a burst effect nearing 100% release (P < 0.05, Figure 9) (31). In contrast, MMT-containing nanoparticles demonstrated sustained VAN release over 21 days (P < 0.05), significantly enhancing the release profile (Table 6). The sustained 21-day release system holds clinical significance for peri-implantitis treatment by providing continuous therapeutic levels during the critical moment to control inflammation and bacterial colonization, thereby preventing disease progression. The study has shown that biweekly administration of sustained-release formulations can markedly reduce peri-implantitis progression within approximately 15 days (62). Drug release kinetics were analyzed using multiple models, including First-order, Higuchi, Hixson-Crowell, and Baker-Lonsdale models.

**Table 6.** Cumulative Drug Release for the Chitosan/Vancomycin and Chitosan-Based Nanocarrier Formulated with Montmorillonite and Vancomycin Nanoparticle Versus Immersion Time ^a^

Variables	Time (h)								
	2	4	8	24	48	74	168	336	504
**Release (%) CS/VAN**	60.33	79.67	81.67	84.33	91.67	100.00	-	-	-
**SD CS/VAN**	4.04	7.09	3.51	6.43	2.52	-	-	-	-
**Release (%) CS/MMT/VAN**	23.33	33.33	43.67	47.00	47.33	51.33	56.67	68.00	73.00
**SD CS/MMT/VAN**	3.57	3.21	4.16	4.78	2.52	1.53	1.95	6.08	2.65

Abbreviations: CS/VAN, chitosan/vancomycin; CS/MMT/VAN, chitosan-based nanocarrier formulated with montmorillonite and vancomycin.

^a^ The experiments were done in triplicate.

In this study, the Higuchi model showed a strong fit with an R^2^ value of 0.935, indicating that the drug release mechanism predominantly follows diffusion-controlled kinetics, where drug release is proportional to the square root of time. This suggests that VAN is mainly released from the nanoparticles via Fickian diffusion, consistent with typical matrix-based delivery systems (63-65). In contrast, the Korsmeyer-Peppas model exhibited a poor fit (R^2^ = 0.193), yet the release exponent (n) was 0.931. According to the Korsmeyer-Peppas model, an n (n = 0.931) slightly above 0.89 suggests super case II transport, implying that drug release is governed not only by diffusion but also significantly influenced by polymer relaxation or swelling mechanisms. This indicates a complex release process involving both diffusion and polymer matrix relaxation (58). Despite this mechanistic insight, the geometry and assumptions of the Korsmeyer-Peppas model did not adequately fit the data, whereas the superior statistical fit of the Higuchi model reinforces diffusion as the dominant release mechanism in these nanoparticles (Table 7). 

**Table 7.** Release Kinetic Coefficients for Optimized Chitosan-Based Nanocarrier Formulated with Montmorillonite and Vancomycin Nanoparticle

Models	Optimized CS/MMT/VAN
**First order**	
K	0.003
R^2^	0.909
**Higushi**	
k	2.470
R^2^	0.935
**Hixson-Crowell**	
K	-0.007
R^2^	0.859
**Korsmeyer-Peppas**	
N	0.931
R^2^	0.193
**Baker-Lonsdale**	
R^2^	0.0996

Abbreviation: CS/MMT/VAN, chitosan-based nanocarrier formulated with montmorillonite and vancomycin.

### 4.11. Antimicrobial Activity

To validate the antimicrobial efficacy of the in vitro release media against gram-positive *S. aureus*, inhibition zone sizes were measured and statistically compared with positive controls (VAN and chlorhexidine). As presented in Table 8, the CS/MMT/VAN formulation produced significantly larger inhibition zones at 6 hours (13.7 ± 0.01 mm) compared to VAN (11.3 ± 0.01 mm) and chlorhexidine (5.17 ± 0.02 mm, P < 0.05, Table 8), indicating enhanced initial antibacterial activity. Although the inhibition zones decreased at 24 hours and 21 days, they remained comparable or superior to controls, demonstrating sustained antimicrobial effects.

**Table 8.** Effect of in vitro Release Media (6 h, 24 h, 21 days) Against *Staphylococcus aureus* ^a, b, c^

Groups	Inhibition Zone of *Staphylococcus aureus* (mm, h)		
	6	24	21
**(+) control (VAN)**	11.3 ± 0.01		
**(+) control (chlorohexidine)**	5.17 ± 0.02		
**(-) control (blank)**	-		
**CS/MMT/VAN**	13.7 ± 0.01	10.7 ± 0.01	9.6 ± 0.01

Abbreviations: VAN, vancomycin; CS/MMT/VAN, chitosan-based nanocarrier formulated with montmorillonite and vancomycin.

^a^ Inhibition zone of *Staphylococcus aureus* (mm).

^b^ The experiments were done in triplicate (n = 3).

^c^ The values are expressed as mean ± SD.

The MIC of VAN against *S. aureus* was determined to be 0.25 mg/L for growth inhibition, with 0.75 mg/L required for complete prevention of colonization. Importantly, the drug concentrations released at all tested time points from the nanoparticles exceeded these MIC thresholds, confirming the release system's capability to maintain therapeutic drug levels. This indicates the potential clinical relevance of the developed CS/MMT/VAN formulation in delivering sustained antibacterial action without exceeding toxic concentrations. It is important to mention that antimicrobial agents at sub-MIC levels have an inductive effect on biofilm development and may lead to possible risks of bacterial resistance (66).

### 4.12. Evaluating the Cytotoxicity of a Nanoparticle-In-Situ Gel

**Figure 10.**
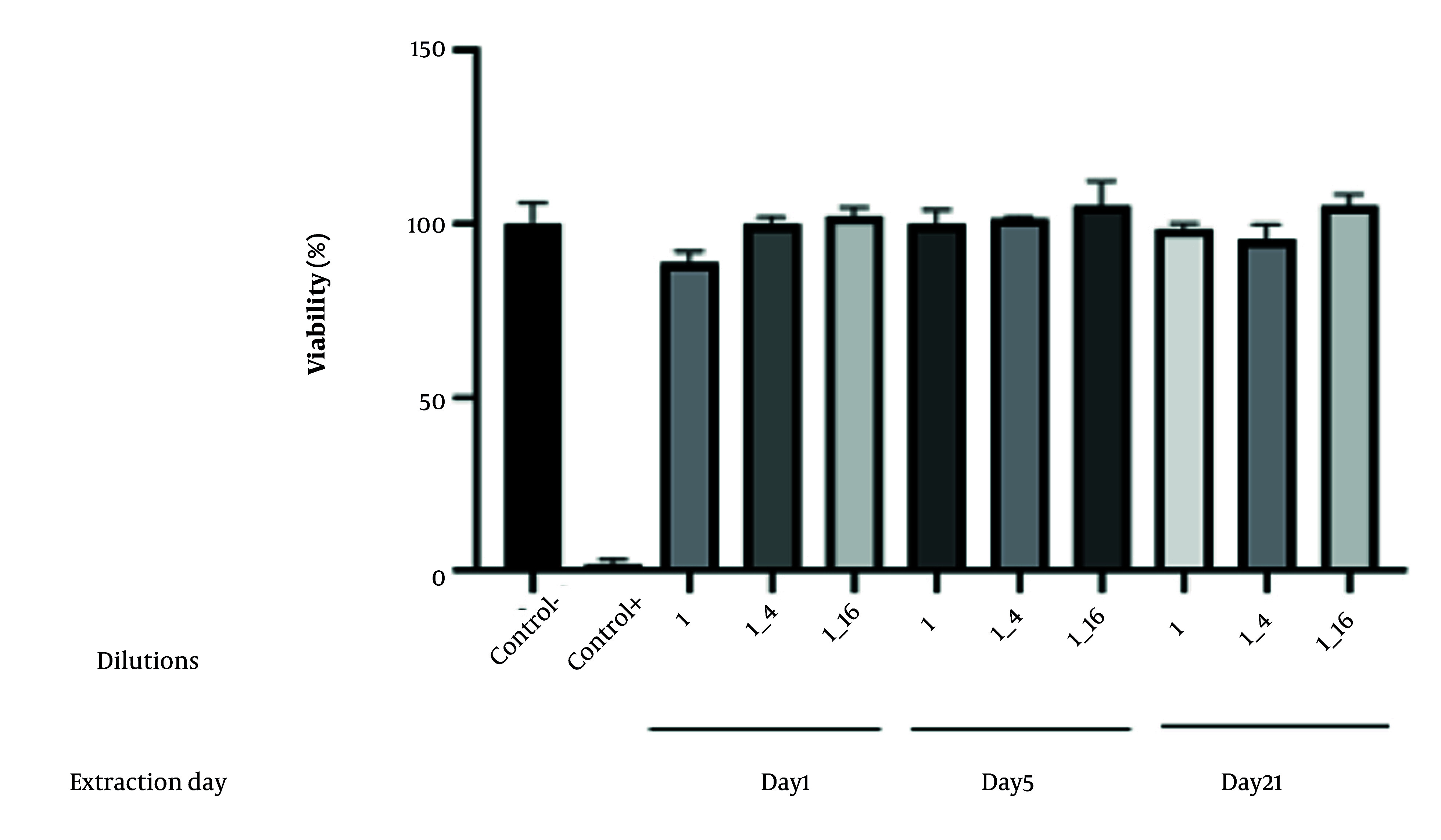
The cytotoxicity of a nanoparticle-in-situ gel

The cytotoxic potential of the optimized CS/VAN/MMT nanoparticles was evaluated by assessing cell viability at three extraction time points (day 1, 5, and 21) according to ISO 10993-5 guidelines (Figure 10). Cell viability was expressed as a percentage relative to the negative control, with mean values and standard deviations reported for each condition. The mean cell viability percentages were 100.24 ± 6.06% for Day 1, 89.21 ± 3.03% for day 5, and 100.24 ± 2.00% for day 21 extracts. According to ISO 10993-5 criteria, viability levels of 70% or greater indicate non-cytotoxicity. All tested extracts exceeded this threshold, demonstrating no cytotoxic effects under the conditions employed. These findings suggest the nanoparticles do not adversely affect cell metabolic activity or membrane integrity, supporting their biocompatibility. The observed variability, as shown by the standard deviations, was within acceptable limits and did not impact the overall cytotoxicity assessment.

### 4.13. Conclusions

The study explored the CS/MMT nanoparticle for the sustained release of VAN. The incorporation of MMT nano clay into the CS framework enhanced stability and extended drug diffusion. Utilizing electrospraying has produced spherical, drug-loaded CS/MMT nanospheres at the nanoscale. These results show potential as a localized sustained delivery system; further in vivo studies are warranted.

### 4.14. Limitations

This study lacks evaluation in relevant in vivo or ex vivo peri-implant tissue models, which are critical for assessing biological responses under physiological conditions. Additionally, long-term stability testing of the nanoparticles and gels was not performed, limiting understanding of their durability over extended periods. Potential cytotoxicity concerns remain regarding the relatively high acetic acid concentration used in the formulation, warranting further investigation.

### 4.15. Future Directions

Future work should focus on in vivo validation of the CS/MMT nanoparticle system to establish clinical efficacy and safety, alongside scaling-up processes for potential clinical translation. It is also essential to utilize more complex biofilm models instead of solely planktonic *S. aureus* to better mimic peri-implantitis microbial communities.

ijpr-24-1-161934.pdf

## Data Availability

All data analyzed during this study are included in this published article and can be accessed by readers beyond just upon request.
